# Nonconscious information can be identified as task-relevant but not prioritized in working memory

**DOI:** 10.1093/cercor/bhac208

**Published:** 2022-06-06

**Authors:** Tiziana Pedale, Aurelie Fontan, Filip Grill, Fredrik Bergström, Johan Eriksson

**Affiliations:** Umeå Center for Functional Brain Imaging, Umeå University, 901 87 Umeå, Sweden; Department of Integrative Medical Biology, Physiology Section, Umeå University, 901 87 Umeå, Sweden; Umeå Center for Functional Brain Imaging, Umeå University, 901 87 Umeå, Sweden; Department of Integrative Medical Biology, Physiology Section, Umeå University, 901 87 Umeå, Sweden; Umeå Center for Functional Brain Imaging, Umeå University, 901 87 Umeå, Sweden; Department of Radiation Sciences, Diagnostic Radiology, Umeå University, 901 87 Umeå, Sweden; CINEICC, Faculty of Psychology and Educational Sciences, University of Coimbra, Rua do Colégio Novo, 3001-802 Coimbra, Portugal; Umeå Center for Functional Brain Imaging, Umeå University, 901 87 Umeå, Sweden; Department of Integrative Medical Biology, Physiology Section, Umeå University, 901 87 Umeå, Sweden

**Keywords:** consciousness, attention, distraction, endogenous control, fMRI

## Abstract

Two critical features of working memory are the identification and appropriate use of task-relevant information while avoiding distraction. Here, in 3 experiments, we explored if these features can be achieved also for nonconscious stimuli. Participants performed a delayed match-to-sample task in which task relevance of 2 competing stimuli was indicated by a cue, and continuous flash suppression was used to manipulate the conscious/nonconscious visual experience. Experiment 1 revealed better-than-chance performance with nonconscious stimuli, demonstrating goal-directed use of nonconscious task-relevant information. Experiment 2 demonstrated that the cue that defined task relevance must be conscious to allow such goal-directed use. In Experiment 3, multi-voxel pattern analyses of brain activity revealed that only the target was prioritized and maintained during conscious trials. Conversely, during nonconscious trials, both target and distractor were maintained. However, decoding of task relevance during the probe/test phase demonstrated identification of both target and distractor information. These results show that identification of task-relevant information can operate also on nonconscious material. However, they do not support the prioritization of nonconscious task-relevant information, thus suggesting a mismatch in the attentional mechanisms involved during conscious and nonconscious working memory.

## Introduction

Working memory is the temporary retention of relevant information for prospective use (Fuster 1995, 2009; Baddeley 2010), which is crucial for many domains of cognition such as planning, problem-solving, language, and more. Traditionally, working memory was considered to require a conscious experience of the memorandum. However, several recent studies have demonstrated that nonconscious information can be maintained for short periods (Soto et al. 2011; Bergström and Eriksson 2014, 2015; King et al. 2016; Trübutschek et al. 2017; Bergström and Eriksson 2018; Trübutschek et al. 2019a, 2019b), suggesting that nonconscious information can be maintained in working memory (Soto and Silvanto 2014). The maintenance of information over a short delay is the most basic feature of working memory, but the concept also includes “executive” components, among which a key role is exerted by top-down attention (Fuster 2009; Gazzaley and Nobre 2012; Eriksson et al. 2015). Indeed, a critical feature of working memory is that it serves endogenous goal-directed behavior (Fuster and Bressler 2012), requiring the capacity to identify task-relevant information for prospective use and to avoid being distracted by irrelevant stimuli (Zanto and Gazzaley 2009). Thus, a key question is whether goal-directed use of task-relevant information can occur with nonconsciously presented stimuli. Moreover, it is not known whether the endogenous goal-directed use of nonconscious information would be the result of selective prioritization of the task-relevant information and filtering of the distractor.

Previous studies have demonstrated that attention can affect both conscious and nonconscious stimulus processing (McCormick 1997; Jiang et al. 2006; Huang et al. 2014; Herreros et al. 2017), even when the cue that directs attention is presented nonconsciously (Kentridge et al. 1999). Moreover, Pan and colleagues (Pan et al. 2014) found that nonconsciously presented information was retained in memory only if it was task-relevant, as revealed by its biasing effects on visual perception of a second stimulus. Considering these previous findings, it seems likely that goal-directed use and selective prioritization of task-relevant information to support a delayed response-decision could occur on nonconsciously presented information. We here investigate this issue in 2 behavioral experiments and one functional magnetic resonance imaging (fMRI) experiment using a delayed match-to-sample task with conscious and nonconscious stimuli. In all experiments, the relevance of the stimuli was defined on a trial-by-trial basis by a cue, which was conscious in Experiments 1 and 3, and nonconscious in Experiment 2. The cue indicated which of 2 shapes, a circle or a diamond, was the target on a particular trial. The task consisted of remembering the target’s location and, after a delay, answering whether a probe pointed to the location of the target or not. If both shapes are passively stored, then there should be recognition of any probe matching the location of 1 of the 2 shapes, irrespectively of their status as target/distractor. Alternatively, if the target shape is discriminated from the distractor shape, and this information is used for goal-directed purposes, there should be more hits to probes matching the target location than false alarms to probes matching the distractor location. In the fMRI experiment, multi-voxel pattern analyses (MVPA) were used to investigate whether attentional prioritization to the target was implemented to solve the task, and whether it was contingent on the conscious or nonconscious nature of the stimuli. Since previous research has demonstrated that attention directed towards the left or right visual fields is reflected in a difference in brain activity across hemispheres (Vogel et al. 2005; Kuo et al. 2009; Simpson et al. 2011; Vossel et al. 2012), we expected that selective prioritization and retention of the target should manifest as distinguishable patterns of brain activity that toggles in accordance with the target’s location (right or left). We therefore used MVPAs to evaluate if the location of the target could be decoded in regions related to visuospatial processing when the stimuli were consciously or nonconsciously presented.

## Experiment 1

In Experiment 1, we investigated the identification and goal-directed use of nonconscious task-relevant information after the presentation of a conscious cue that indicates which of 2 shapes, a target or a diamond, was the target. After the cue, the target and distractor were presented in separate quadrants of the visual display. Continuous flash suppression (CFS) was used to manipulate the visual experience of the target and distractor (see below). The task consisted of remembering the target’s location for a 5–15 s delay, after which the participants had to answer whether a probe pointed to the location of the target or not (yes or no).

### Materials and methods

#### Participants

In total, 32 participants took part in Experiment 1 (17 females, mean age ± standard deviation, SD: 27.3 ± 4.7 years old). All participants had normal or corrected to normal vision, were right eye-dominant, right-handed, and reported not to be affected by colorblindness or any neurological or psychiatric disease. They gave written informed consent and were paid 300 SEK for participation. The study was approved by the regional ethics review board (2017-77-31M).

#### Experimental design

Stimuli presentations and response collection were accomplished through E-Prime 2.0. Participation consisted of attending 2 occasions occurring within 7 days: training and the actual experiment. The training was constituted by a short version of the task (78 trials) with the purpose to ensure that each participant understood the task and that the CFS manipulation worked as intended (see below). The actual experiment consisted of 312 delayed match-to-sample trials divided into 3 conditions: 64 conscious, 224 nonconscious, and 24 absent trials, performed in 4 blocks (78 trials each) to allow the participants to take breaks. Each trial was drawn randomly from the 3 conditions and began with an intertrial interval (ITI; 3 s) before the cue presentation. The cue was either a circle or a diamond shape presented in the center of the screen for 1.75 s on a green background, and indicated which shape was relevant on a trial-by-trial basis. Immediately after the cue presentation, a gray screen was displayed (0.25 s), followed by the sample presentation (1 s). The sample consisted of a gray silhouette of the cue shape (the target) presented together with a nonrelevant shape (the distractor). Thus, if the circle was the defined target, the diamond acted as a distractor for that trial, and vice versa. The target was presented in an equal amount of trials in each of the 4 quadrants of the screen and the task consisted of remembering its location. The distractor could appear in 1 of the 2 quadrants located on the opposite side (right or left).

We used CFS to manipulate the visual experience of the memory sample. A mirror stereoscope was used to isolate visual input from one side of the screen to the participant’s corresponding eye. In the conscious condition, the sample was presented to the dominant (right) eye and the gray shapes (RGB, i.e., red-green-blue = 198, 198, and 198) were superimposed on colored squares of random composition (Mondrians) that were flashed (10 Hz) to the same eye. In the nonconscious condition, the sample consisted of the gray shapes on a gray background (RGB = 210, 210, and 210) presented only to the nondominant (left) eye, whereas Mondrians were flashed to the dominant eye. This procedure suppressed the sample from conscious experience (Tsuchiya and Koch 2005). During the nonconscious trials, the sample was presented for 500 ms, whereas the Mondrians were flashed for 1 s to minimize the risk of adaptation after-effects (Tsuchiya and Koch 2005). During the absent trials, Mondrians were presented to the dominant eye, whereas an empty gray background (RGB = 210, 210, and 210) was presented to the nondominant eye. Importantly, unless the sample stimuli broke suppression, nonconscious and absent trials led to the same visual experience (experiencing only Mondrians). These “absent” trials served as a reference condition for the nonconscious trials, such that the subjective visual experience was identical for nonconscious and absent trials (seeing only Mondrians), and any response bias could be estimated independently from target and distractor effects (i.e. the tendency to respond “no” more often than “yes” when no target and distractor was seen).

After a delay phase (5–15 s) consisting of a gray screen with a central white dot, a memory probe was presented. The probe consisted of a “clock pointer” aimed at 1 of the 4 quadrants. The task consisted of recognizing if the probe was pointing to the quadrant of the target (yes/no response). If participants did not visually experience a target stimulus (i.e. only experienced Mondrians) they were instructed to guess according to their gut feeling/first alternative that came to mind at the appearance of the probe. The participants had a maximum time of 5 s to answer, after which the next trial was initiated. The chance for the probe to point to the target position was 50%. For the conscious condition, the probe could point to the correct target location (“target-match”), to the distractor location (“distractor-match”), or to an empty location (“non-match”). In the nonconscious condition, the probe could indicate only the correct target location (“target-match”) or the distractor location (“distractor-match”), to maximize power for the comparison of hits vs. “distractor-match” false alarms. For conscious trials, there were 32 “target-match” probes, 16 “non-match” probes, and 16 “distractor-match” probes. For nonconscious trials, there were 112 “target-match” probes and 112 “distractor-match” probes.

**Fig. 1:**
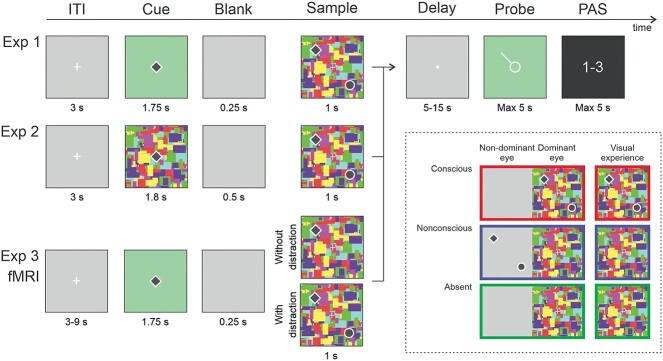
Sequence of events for the trials of the delayed match-to-sample working memory tasks of the 3 experiments. In all the 3 experiments, the cue was either a diamond or a circle. The images inside the quadrant with dash borders schematically represent the CFS manipulation in the 3 presentation conditions (conscious, nonconscious, and absent). In the conscious condition the shapes were presented to the dominant eye (right eye), in the nonconscious condition the shapes were presented to the nondominant eye (left eye), and in the absent condition an empty background was presented to the nondominant eye. For each of the 3 conditions, Mondrian were displayed to the dominant eye. The last column in the quadrant with dash borders displays the visual experience of participants. Note that for the purpose of illustration only, the shapes are contoured in white.

At the end of each trial, participants were asked to report their perception of the sample on a modified (3-point) perceptual awareness scale (PAS; Ramsøy and Overgaard 2004), ranging from no perceptual experience (PAS = 1), vague perceptual experience (PAS = 2), and clear perceptual experience (PAS = 3; for a similar approach, see, Christensen et al. 2006; Bergström and Eriksson 2018; Fontan et al. 2021). The PAS prompt was presented for a maximum of 5 s. See Fig. 1 for illustration of the task.

The contrast of the sample shapes during the nonconscious trials was adjusted every 10 nonconscious trials based on the PAS response, ensuring that participants did not break the suppression for at least 70% of nonconscious trials. Specifically, if the participants reported some experience of the shapes (PAS > 1) > 3 out of 10 nonconscious trials, the contrast between the shapes and the background decreased (thus making harder the processing of the nonconscious stimuli), otherwise, it increased (thus making the processing of the shapes easier). This adjustment was done to maximize the generally weak processing of the nonconscious stimuli. Notably this contrast adjustment procedure is independent of task performance, since the adjustment is based solely on the visibility ratings. Each contrast value consisted of a 2-point increase/decrease in RGB value of the gray shapes (range = 206–182) relative to the gray background (RGB = 210, 210, and 210). Each participant started the training with a contrast value of RGB = 196, 196, and 196, and started each subsequent block with the last contrast value reached in the previous block.

#### Statistical analysis

Trials with response time (RT) of < 250 ms or with no response were excluded before statistical analyses (Ratcliff 1993). Afterwards, only trials in absent and nonconscious conditions with PAS = 1, and trials with PAS = 3 in the conscious condition were included in the following analyses. For completeness, the analyses of the conscious condition were repeated including also the vaguely seen trials (PAS = 2). For the analyses of accuracy, a hit was defined as a “yes” response to a “target-match” probe, whereas a “yes” response to a “distractor-match” probe or to a “non-match” probe was defined as a false alarm. All statistical tests are 2-tailed. To further substantiate any null result, we report also the Bayes factor (BF_01_) according to the following interpretation: BF_01_ < 1: no evidence, 1–3: anecdotal evidence, 3–10: substantial evidence, 10–30: strong evidence, 30–100: very strong evidence, and > 100: decisive evidence (Keysers et al. 2020).

### Results

All trials with PAS > 1 were removed from the nonconscious (mean ± SD: 28.9 ± 10.7%) and absent conditions (10.2 ± 12.8%) to ensure no visual experience of the target, and all trials with PAS < 3 were removed from the conscious condition (6.8 ± 10.9%; see Supplementary Table 1).

As described in the introduction, the task consisted of remembering the target’s location and, after a delay, answering whether a probe pointed to the location of the target or not. If both shapes are passively stored without discrimination of task-relevant information (i.e. target/distractor), then there should be recognition of any probe matching the location of 1 of the 2 shapes, irrespectively of their status as target/distractor. Alternatively, if the target shape is discriminated from the distractor shape, and this information is used for goal-directed purposes, there should be more hits to probes matching the target location than false alarms to probes matching the distractor location.

**Fig. 2:**
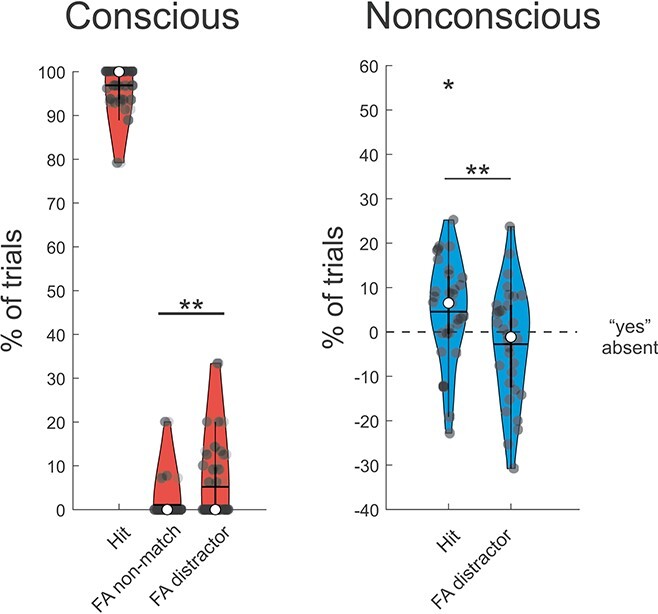
Behavioral performance for the working-memory task in Experiment 1 in the conscious, and nonconscious conditions. The violin plots show the percentage of hits when the probe pointed towards the correct location (Hit), the percentage of false alarms when the probe pointed towards an empty position (FA non-match), and the percentage of false alarms when the probe pointed towards the distractor’s position (FA distractor), in the conscious (red), and nonconscious (blue) conditions. Note that the gray dashed line in the nonconscious condition represents the chance level when adjusted for the rate of false alarms in the absent condition (i.e. from each nonconscious condition the percentage of “yes”_absent_ was subtracted). In all violin plots, median, and mean are represented by the white dot and the horizontal black line, respectively. ^*^P < 0.05, ^**^P < 0.01, and ^***^P < 0.001.

For conscious trials, performance was 93.6 ± 8.9% (mean ± SD for hits—false alarms). Despite the high level of performance there was evidence for distraction, such that there were significantly more false alarms when the probe pointed towards the position of the distractor (5.2 ± 8.2%) compared with an empty position (1.1 ± 3.9%; Wilcoxon matched-pairs test: *Z* = 2.67, *P* = 0.0076; see Fig. 2). These results remained when including vaguely seen conscious trials (i.e. PAS = 2): mean performance was 93.3 ± 8.9%; and there were significantly more false alarms in presence of probes pointing towards the position of the distractor compared with an empty position (Wilcoxon matched-pairs test: *Z* = 2.67, *P* = 0.008).

Crucially, performance was also significant for nonconscious trials (hits—false alarms: 7.3 ± 11.6%; *t*_31_ = 3.56, *P* = 0.0012; see Fig. 2, and Supplementary Fig. 1), demonstrating that identification and goal-directed use of task-relevant information can be performed for nonconsciously presented stimuli. Moreover, when compared with the rate of “yes” responses in the absent condition (“yes”_absent_), which constitutes the information-free guessing rate for each individual, the hits rate was significantly different from the guessing rate (hits—“yes”_absent_: 4.6 ± 11.6%; *t*_31_ = 2.23, *P* = 0.033; see Fig. 2, and Supplementary Fig. 1). On the contrary, the false alarm rate was not significantly different from the guessing rate (false alarms—“yes”_absent_: −2.8 ± 12.7%; *t*_31_ = −1.22, *P* = 0.24, BF_01_ = 2.68; see Fig. 2, and Supplementary Fig. 1). These results suggest that, unlike for conscious trials, the distractor did not cause significant interference. According to the BF the strength of the evidence supporting a zero effect was weak, although it is noteworthy that the direction of the effect was opposite to distraction (i.e. the false alarm rate was nominally *lower* than the guessing rate).

## Experiment 2

The aim of Experiment 2 was to investigate whether the assignment of task relevance to nonconscious stimuli that was evident in Experiment 1 depends on conscious processing of the cue, or if cue-initiated deployment of attention could be performed nonconsciously. Thus, the design of the second experiment was identical to Experiment 1 except for the cue phase.

### Materials and methods

#### Participants

In total, 27 participants took part in Experiment 2. One of them was not included in the analyses because of failing to follow task instructions. Thus, 26 participants were included in the analyses of Experiment 2 (19 females, 26.5 ± 5.8 years old). All participants had normal or corrected to normal vision, were right eye-dominant, right-handed, and reported not to be affected by colorblindness or any neurological or psychiatric disease. They gave written informed consent and were paid 300 SEK for participation. The study was approved by the regional ethics review board (2017-77-31M).

#### Experimental design

Experiment 2 was identical to Experiment 1 except for the cue visibility, which was also manipulated. In the conscious condition, the cue (1.8 s) and the sample (1 s) were presented to the dominant eye, superimposed on the Mondrians, and therefore fully visible. In the nonconscious condition, both the cue and the sample were presented to the nondominant eye (i.e. suppressed). The cue was presented for 1.3 s, whereas the Mondrians were flashed for 1.8 s. Similarly, the nonconscious sample was presented for 500 ms, whereas the Mondrians were flashed for 1 s. That is, in the cue and sample phases, the flashing of the Mondrian lasted for 500-ms longer, to minimize the risk of adaptation after-effects. To clearly separate the cue phase from the sample phase, the duration of the gray screen between the 2 phases was 500 ms. During the absent trials, Mondrians were presented to the dominant eye during the cue and the sample phase, whereas an empty gray background was presented to the nondominant eye during both phases, leading to the same visual experience as in nonconscious trials (i.e. no experience of the cue and the sample). Participants were instructed to use the PAS response to reflect their visual experience of both the cue and the sample stimuli, such that the PAS rating would reflect the most intense experience of the stimuli. See Fig. 1 for an illustration of the task.

#### Statistical analysis

Trials with RT of < 250 ms or with no response were excluded before statistical analyses (Ratcliff 1993). Afterwards, only trials in absent and nonconscious conditions with PAS = 1, and trials with PAS = 3 in the conscious condition were included in the following analyses. As for Experiment 1, the analyses of the conscious condition were repeated including also the vaguely seen trials (PAS = 2). For the analyses of accuracy, a hit was defined as a “yes” response to a “target-match” probe, whereas a “yes” response to a “distractor-match” probe or to a “non-match” probe was defined as a false alarm. All statistical tests are 2-tailed.

### Results

All trials with PAS > 1 in the nonconscious and the absent conditions (mean ± SD: 29.4 ± 13.6%, and 11.4 ± 14.2%, respectively) were excluded from further analyses. Moreover, all trials with PAS < 3 in the conscious condition (4.6 ± 6.2%) were removed from the conscious condition (see Supplementary Table 1).

**Fig. 3:**
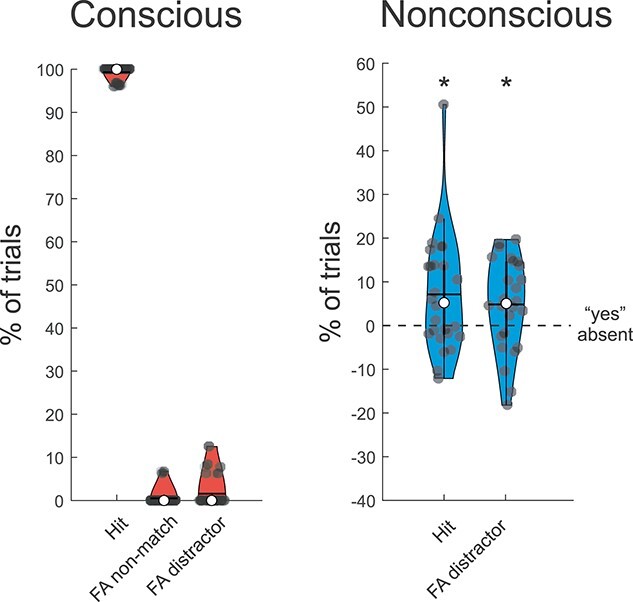
Behavioral performance for the working-memory task in Experiment 2 in the conscious, and nonconscious conditions. The violin plots show the percentage of hits when the probe pointed towards the correct location (Hit), the percentage of false alarms when the probe pointed towards an empty position (FA non-match), and the percentage of false alarms when the probe pointed towards the distractor’s position (FA distractor), in the conscious (red), and nonconscious (blue) conditions. Note that the gray dashed line in the nonconscious condition represents the chance level when adjusted for the rate of false alarms in the absent condition (i.e. from each nonconscious condition the percentage of “yes”_absent_ was subtracted). In all violin plots, median and mean are represented by the white dot and the horizontal black line, respectively. ^*^P < 0.05, ^**^P < 0.01, and ^***^P < 0.001.

As in Experiment 1, performance was very high during conscious trials (hits—false alarms: 98.3 ± 2.9%), but in contrast to Experiment 1 there were not more false alarms when the probe pointed towards the position of the distractor (1.6 ± 3.5%) compared with an empty position (0.5 ± 1.8%; Wilcoxon matched-pairs test: *Z* = 1.47, *P* = 0.14, BF_01_ = 3.11; see Fig. 3). The analyses including vaguely seen conscious trials (i.e. PAS = 2) showed similar results: mean performance was 97.2 ± 4.5% and there was no significant difference between non-match probes and distractor-match probes, although with anecdotal evidence against this effect (Wilcoxon matched-pairs test: *Z* = 1.78, *P* = 0.075, BF_01_ = 2.41).

For nonconscious trials, there were not significantly more hits than false alarms (hits—false alarms: 2.3 ± 12.5%; *t*_25_ = 0.94, *P* = 0.36, BF_01_ = 3.24), suggesting substantial evidence against the identification of the task-relevant shape when participants were unaware of the cues. However, there were significantly more nonconscious hits than “yes” responses during the absent condition (hits—“yes”_absent_: 7.1 ± 13.3%; *t*_25_ = 2.72, *P* = 0.012), as well as more false alarms than “yes” responses during the absent condition (false alarms—“yes”_absent_: 4.8 ± 10.5%; *t*_25_ = 2.35, *P* = 0.027; see Fig. 3, and Supplementary Fig. 1). Thus, the position of both the target and distractor could be remembered when presented nonconsciously, but it was not possible to differentiate between task-relevant and distracting information when the cue that defined relevance was presented nonconsciously.

## Experiment 3

In Experiment 3, we used fMRI to investigate which mechanisms are implemented to allow the goal-directed use of task-relevant information demonstrated in Experiment 1. In particular, we believed that the investigation of these mechanisms could explain why there was no distractor interference in the nonconscious condition. Possibly, the task is solved through selective prioritization of the target location that would for some reason be stronger for nonconscious (no interference) than conscious (significant interference) trials. Alternatively, successful task performance could result from encoding and maintenance of both target and distractor, where information of the distractor is used in a goal-directed way to minimize false alarms. To address these questions, we used a similar design as in Experiment 1, but we added conditions where the target was presented alone (consciously or nonconsciously), i.e. “without the distractor” condition. On the basis of previous researches demonstrating that attention directed towards the left or right visual fields is reflected in a difference in brain activity across the hemispheres (Vogel et al. 2005; Kuo et al. 2009; Simpson et al. 2011; Vossel et al. 2012), we used MVPA to evaluate if the location of the target could be decoded in regions related to visuospatial processing. However, if the locations of both the target and the distractor are encoded and maintained, the left vs. right difference should be attenuated or even absent, and there should be a significant difference in brain activity between the conditions “with” vs. “without the distractor” (encoding and maintaining 1 vs. 2 items). If instead the distractor is filtered out, we would expect no better-than-chance decoding of the “with” vs. “without the distractor” conditions because of the selective focusing to the location of the target in both conditions, although this may be clearer during the maintenance rather than during the sample phase (i.e. the stimulus presentation), where perceptual processes are also driving brain activity (seeing 1 vs. 2 items in the display).

### Materials and methods

#### Participants

In total, 31 participants took part in Experiment 3. One of the participants was not included in the analyses because of failing to follow task instructions. Thus, 30 participants were included in the analyses of Experiment 3 (18 females, 28.2 ± 4.6 years old). All participants had normal or corrected to normal vision, were right eye-dominant, right-handed, and reported not to be affected by colorblindness or any neurological or psychiatric disease. They gave written informed consent and were paid 500 SEK for participation. The study was approved by the regional ethics review board (2017-77-31M).

#### Experimental design

The task used for the fMRI study was the same as in Experiment 1 except for the following modifications (see Fig. 1 for illustration of the task). A “without distraction” condition was added in which the target was presented alone. Also, when present, the distractor could appear in 1 of the 3 empty quadrants (“with distraction” condition). There was no condition in which the distractor was presented alone (without the target). The fMRI task consisted of 222 delayed match-to-sample trials divided into 3 conditions: 60 conscious (36 with and 24 without a distractor), 120 nonconscious (72 with and 48 without distractor), and 42 absent trials, divided into 3 runs (74 trials each). For conscious trials with a distractor, there were 18 “target-match” probes, 9 “non-match” probes, and 9 “distractor-match” probes. For conscious trials without a distractor, there were 12 “target-match” probes, and 12 “non-match” probes. For nonconscious trials with distractor, there were 36 “target-match” probes and 36 “distractor-match” probes. For nonconscious trials without a distractor, there were 24 “target-match” probes and 24 “non-match” probes. The sequence and timing of events for each trial were the same as in Experiment 1 except for the ITI (now 3–9 s). In addition, the proportion of nonconscious trials with PAS = 1 was increased to 80%. That is, the contrast value decreased if the participants reported some experience of the shapes (PAS > 1) in > 3 out of 10 nonconscious trials. Finally, the range of RGB value, which defines the stimuli contrast, was extended (206–174).

The MRI data were collected with a GE 3 Tesla Discovery MR750 scanner (32-channel receive-only head coil). Each participant underwent 1 fMRI session with 3 functional runs (810 volumes each) of scanning using a *T*2*-weighted gradient echo pulse sequence, echo planar imaging, field of view = 25 cm, matrix size = 96 × 96, slice thickness = 3.4 mm, 37 slices with no interslice skip and an ASSET acceleration factor of 2. The volumes covered the whole cerebrum and most of the cerebellum. The acquisition orientation was oblique axial and aligned with the anterior and posterior commissures, and the slices were acquired in interleaved order with time echo (TE) = 30 ms, time repetition (TR) = 2 s, and flip angle = 80°. Between the first and the second functional runs a high-resolution *T*1-weighted structural image was collected with Fast Spoiled Gradient Echo (FSPGR) with TE = 3.2 ms, TR = 8.2 ms, TI = 450 ms, and flip angle = 12°.

### Data processing and statistical analysis

#### Behavioral statistical analysis

Trials with RT of < 250 ms or with no response were excluded before statistical analyses (Ratcliff 1993). Afterwards, only trials in absent and nonconscious conditions with PAS = 1, and trials with PAS = 3 in the conscious condition were included in the following analyses. As for Experiments 1 and 2, the analyses of the conscious condition were repeated including also the vaguely seen trials (PAS = 2). For the analyses of accuracy, a hit was defined as a “yes” response to a “target-match” probe, whereas a “yes” response to a “distractor-match” probe or to a “non-match” probe was defined as a false alarm. All statistical tests are 2-tailed.

#### Preprocessing and intra-subject General Linear Model

Image preprocessing and intra-subject modeling was conducted with SPM12 (Wellcome Department of Imaging Neuroscience, London, United Kingdom) running in Matlab 8.4 environment (Mathworks, Inc., Sherbon, MA, United States) using custom-made Matlab scripts for batching. All images were (i) slice-time corrected using the first slice as a reference, (ii) corrected for head movements between image volumes, (iii) unwarped to remove residual movement-related variance (Andersson et al. 2001), and (iv) co-registered to high-resolution structural data. The structural images were normalized to the Montreal Neurological Institute template using DARTEL (Ashburner 2007) and the resulting parameters were used for normalization of the functional images, which were resampled to 2-mm isotropic voxel size. Finally, the functional images were smoothed with an 8-mm FWHM Gaussian kernel for the univariate analyses, and with a 2-mm FWHM Gaussian kernel for the multivariate pattern analyses (Gardumi et al. 2016).

For intra-subject modeling, a General Linear Model (GLM) was used. The model consisted of the following regressors of interest: presentation conditions (conscious with PAS = 3, nonconscious with PAS = 1, and absent with PAS = 1) for each trial phase (sample, delay, and probe). The regressors of interest related to the sample and delay phase of the conscious and nonconscious trials were divided by target side (left or right) and presence or absence of the distractor. The regressors of interest related to the probe phase were divided by the response category (hits and misses for “target-match,” false alarms, and correct rejections for “distractor-match” and “non-match” probes). The regressors related to the delay phase were modeled with their respective durations, and the sample and probe phase with zero duration. The model also included the following nuisance regressors: irrelevant presentation conditions (conscious with PAS < 3, nonconscious with PAS > 1, and absent with PAS > 1) and missed responses by trial phase (sample, delay, and probe). Head motion (6 parameters) was included as covariates of no interest. All regressors except for head motion were convolved with the SPM12 canonical hemodynamic response function. A high-pass filter (cutoff = 128 s) was applied to remove low-frequency drifts in the data and the autocorrelation model was global AR(1).

#### Univariate analyses

Data were analyzed using a 2-stage summary statistics random effect model (Friston et al. 1995; Holmes and Friston 1998). Contrast maps were computed on beta-maps resulting from the estimated first-level GLMs to reveal for conscious and nonconscious conditions, brain regions subtending visuospatial processing during the sample presentation. Individuals’ maps subtending conscious and nonconscious visuospatial networks were taken to second-level random-effects analyses (1-sample *t*-tests) to account for inter-individual variability. Multiple comparisons correction of statistical maps at the second level was conducted on the whole brain using cluster-based extent thresholding of *P* < 0.05 [family wise error (FWE) corrected] calculated based on the Gaussian random field method and following cluster-defining threshold of *P* < 0.001.

#### Defining regions of interest through searchlight MVPAs

A searchlight decoding approach was used (Kriegeskorte et al. 2006) as implemented in the CoSMoMVPA decoding toolbox (Oosterhof et al. 2016), to identify regions of interest (ROIs) where the mere presence of a stimulus could be decoded. Four searchlights MVPAs were conducted within a restricted brain map including regions involved in visuospatial perception, including occipital-parietal regions and the frontal eye field (Wang et al. 2015, see Fig. 6A) to identify at the individual level the brain regions subtending visuospatial processing of conscious and nonconscious stimuli during the sample and the delay phase (“conscious sample vs. absent sample,” “nonconscious sample vs. absent sample,” “conscious delay vs. absent delay,” and “nonconscious delay vs. absent delay”). We selected this restricted region involved in visuospatial perception because of previous findings on working memory, revealing that maintenance of the content of working memory during a visuospatial task relies mainly on visual and parietal regions (Eriksson et al. 2015; Postle 2016; Bergström and Eriksson 2018; Christophel et al. 2018). Thus, focusing on “visuospatial” regions were expected to be informative for investigating witch kind of information were encoded and maintained during the current task. Furthermore, a searchlight within a more extended map [the perceptual visuospatial mask by Wang et al. (2015) plus the frontal lobe as defined by MNI structural atlas, with the exclusion of the primary motor cortex (bilateral Brodman area 4a and Brodman area 4p) as defined by Juelich histological atlas] was conducted to highlight the brain regions involved in the decoding of the probe phase for conscious and nonconscious trials (“Conscious [with distractor] probe vs. Absent probe”; and “Nonconscious [with distractor] probe vs. Absent probe”; see Fig. 6A). Decoding was performed on the 2-mm smoothed beta parameter maps from the GLM described above (1 map for each trial). The number of maps/trials were balanced for each participant so that in each searchlight analysis there was an equal amount of trials belonging to the conditions that would be of interest in later analyses (left vs. right target side and presence vs. absence of the distractor for the sample and delay phase, and target-match vs. distractor-match for the probe phase). This balancing aimed to avoid any bias in-between conditions.

For each ROI-defining MVPA, we used sphere searchlights (~300 voxels) to extract local features for classification. The searchlight sphere was moved across the voxels belonging to the defined search space (see above). A support vector machine (SVM) was used as a classifier, combined with a 10-fold cross-validation procedure (Varoquaux et al. 2017). As shown by Mumford et al. (2014), within-run cross-validation is unbiased for randomized event-related designs, as used here. Nevertheless, to ensure that blood-oxygen level-dependent (BOLD) signal was nonoverlapping between validation folds, we included only trials such that there was at least 30 s between each adjacent training and test fold, to allow the sluggish BOLD signal to return to baseline. Each map of classification accuracy was smoothed with an 8-mm FWHM Gaussian kernel, and a maximum of 5,000 voxels in the gray matter (as defined by gray matter segmentation using SPM default preprocessing) with the highest accuracy values above chance level (50%) were selected as the individual ROIs (see Fig. 6B). Importantly, the ROI-defining comparisons of conscious/nonconscious vs. absent are orthogonal to the next ROI analyses, except for the decoding of the “Nonconscious delay [without distractor] vs. absent delay” where the ROI defined by the “Conscious delay vs. absent delay” was used to avoid double-dipping effects (Kriegeskorte et al. 2009).

#### ROI analyses

For the ROI analysis, a SVM was used as a classifier, combined with a 10-fold cross-validation procedure and at least 30 s between each adjacent training and test folds. If the number of beta-maps for the 2 conditions of interest were unbalanced, we randomly excluded the exceeding beta-maps until the number of beta-baps was balanced in the 2 conditions. To avoid any bias, we repeated this random exclusion and the subsequent classification 50 times for each subject. The mean of the accuracy values obtained in the 50 iterations was considered as the final individual accuracy value. The mean of the individual accuracy values of each ROI MVPA was entered into a group-level analysis, using a 2-step permutation procedure with 10,000 iterations (Stelzer et al. 2013) to evaluate if the classification at the group level was significantly above chance level (1-tailed). The 2-step permutation procedure consisted of empirically estimating the probability distribution of accuracy separately for each decoding, by applying 10,000 random shufflings of the labels of the 2 conditions involved in each decoding and estimating the corresponding accuracies with the shuffled labels. Then, the probability or significance level for rejecting the null hypothesis is evaluated by comparing the real accuracy against the accumulated empirical distribution. In order to avoid any bias in the accuracy classification for the target side decoding during the sample and delay, the number of trials with/without distractor was balanced across conditions. Similarly, for the decoding of the absence or presence of the distractor during the sample and the delay, the number of trials with left and right targets was balanced across conditions.

### Results

#### Behavioral results

**Fig. 4:**
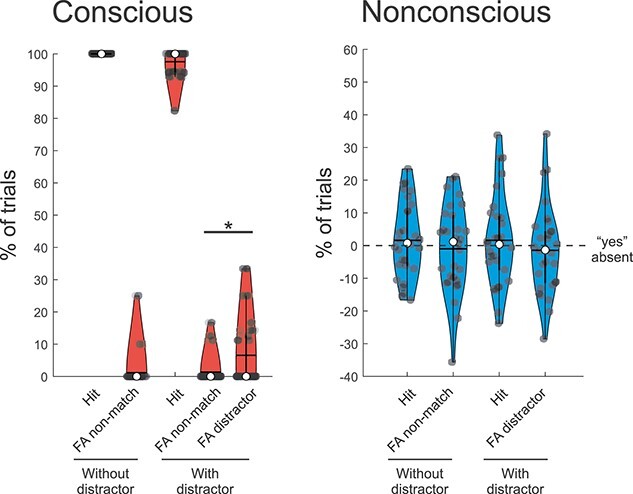
Behavioral performance for the working-memory task in Experiment 3 in the conscious, and nonconscious conditions. The violin plots show the percentage of hits when the probe pointed towards the correct location (Hit), the percentage of false alarms when the probe pointed towards an empty position (FA non-match), and the percentage of false alarms when the probe pointed towards the distractor’s position (FA distractor), in the conscious (red), and nonconscious (blue) conditions. Note that the gray dashed line in the nonconscious condition represents the chance level when adjusted for the rate of false alarms in the absent condition (i.e. from each nonconscious condition the percentage of “yes”_absent_ was subtracted). In all violin plots, median, and mean are represented by the white dot and the horizontal black line, respectively. ^*^P < 0.05, ^**^P < 0.01, and ^***^P < 0.001.

All trials with PAS > 1 in the nonconscious and absent conditions (mean ± SD: 18.3 ± 11.3%, and 9.7 ± 11.1%, respectively), and all trials with PAS < 3 in the conscious condition (6.3 ± 8.1%), were excluded from the main analyses (see Supplementary Table 2). As in the previous 2 experiments, memory performance for conscious trials was very high (hits—false alarms: 98.8 ± 4.9% for the target-only condition, and 93.7 ± 7.5% for the distractor condition), but the presence of distracting information reduced performance significantly (Wilcoxon matched-pairs test: *Z* = 3.10, *P* = 0.002). Moreover, in presence of distracting information, there were significantly more false alarms when the probe pointed towards the position of the distractor (6.6 ± 10.6%) compared with an empty position (1.3 ± 4.2%; Wilcoxon matched-pairs test: *Z* = 2.45, *P* = 0.014; see Fig. 4). Analysis of the conscious trials including vaguely seen trials (i.e. PAS = 2) was also performed. It showed similar results: very high memory performance (98.3 ± 91% for the target-only condition, and 91.8 ± 13.8% for the distractor condition), significant impact of the distracting information (Wilcoxon matched-pairs test: *Z* = 3.43, *P* = 0.0006), and more false alarms in presence of “distractor-match” probes compared with the “non-match” probes (Wilcoxon matched-pairs test: *Z* = 2.59, *P* = 0.010).

For nonconscious trials, performance was not significant for the target-only condition (hits—false alarms: 2.6 ± 17.0%; *t*_29_ = 0.83, *P* = 0.42, BF_01_ = 3.75) or the distractor condition (hits—false alarms: 3.1 ± 12.9%; *t*_29_ = 1.30, *P* = 0.20, BF_01_ = 2.41; see Fig. 4, and Supplementary Fig. 1). The reduced performance in the context of fMRI (Experiment 3) relative to a pure behavioral setting (Experiments 1 and 2) for nonconscious trials is consistent with previous findings (e.g. Bergström and Eriksson 2018), and may be related to the generally more distracting environment during scanning.

#### fMRI results

##### Whole-brain univariate analyses

**Fig. 5:**
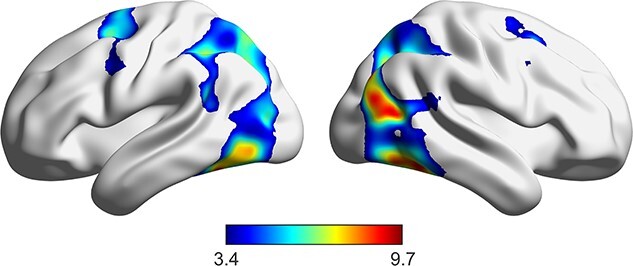
BOLD signal change for Conscious > Absent condition during the sample presentation.

**Fig. 6:**
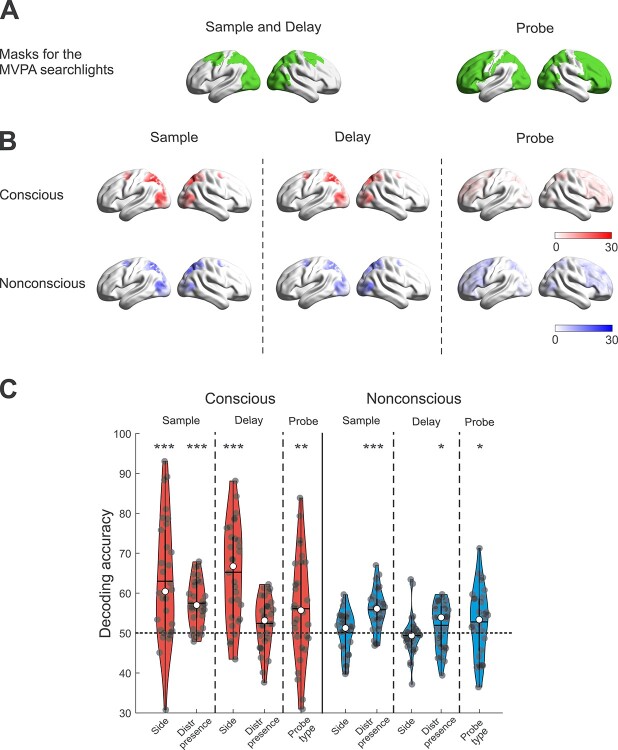
A) Brain masks for the searchlights MVPAs during the 3 phases of the working memory task. B) Brain maps showing the overlap of the individual ROIs in the 30 subjects, separately for each phase of the working memory task, and the conscious/nonconscious conditions. C) Violin plots showing the decoding accuracy for the ROI MVPAs. The possible classifications were: (i) left vs. right target side (Side), (ii) presence vs. absence of the distractor (Distr presence), and (iii) probe pointing towards the position of the target or towards the position of the distractor (Probe type); in the conscious (red) and nonconscious (blue) conditions, during the 3 phases of the working memory task. In all violin plots, median and mean are represented by the white dot and the horizontal black line, respectively. ^*^P < 0.05, ^**^P < 0.01, and ^***^P < 0.001.

We investigated the neural response related to both conscious and nonconscious visuospatial processing during the sample presentation. Whole-brain univariate analyses of fMRI data, contrasting conscious to absent conditions during the sample presentation, revealed significant BOLD signal change in brain areas consistent with visuospatial processing (De Schotten et al. 2005, see Fig. 5). However, as expected these analyses were not sensitive enough to reveal any significant signal change related to nonconscious processing, even at the encoding phase. MVPA is more sensitive compared with univariate analyses (Haxby 2012). We therefore used MVPA to investigate which kind of information were decodable during the sample, the delay and the probe phase of the working memory task with conscious and nonconscious stimuli in the ROIs generated through the searchlights MVPAs (see the Method section above, and Fig. 6B).

#### ROI MVPAs

##### Conscious trials

During the sample phase of the task, the target side (left vs. right) was decoded well above chance level (mean ± SD: 63.0 ± 15.0%; permutation test: *P* < 0.0001; see Fig. 6C), indicating distinguishable patterns of brain activity depending on the side of the target, demonstrating selective prioritization of the target location. However, “with” vs. “without distractor” was also decoded (57.5 ± 5.8%; *P* < 0.0001; see Fig. 6C), suggesting at least perceptual discrimination of the presence or absence of the distractor.

Because the decoding of the target side included also conditions in which the shapes were located on a single side of the screen (i.e. the “without distractor” condition, and the “with distractor” condition when the distractor was presented on the same side as the target) it is possible that the initial decoding of target side was confounded by perceptual processes derived from having visual stimulation located only on one side vs. on both sides of the screen. We, therefore, ran a control analysis in which we excluded the “without distraction condition” and all the trials in the “with distractor” condition in which the shapes were located on a single side. Target side was still decoded in this case (54.3 ± 15.3%, *P* = 0.031).

During the delay phase, target side was again decoded well above chance (65.2 ± 12.0%, *P* < 0.0001). However, only a trend emerged for decoding of the presence vs. absence of the distractor (52.4 ± 6.5%; *P* = 0.06). Thus, brain activity related to the target location but not distractor presence remained significantly decodable during the delay phase, demonstrating that the location of the target was maintained in working memory, whereas there was no clear evidence of maintenance of distractor information, thus suggesting attentional prioritization of the target.

Finally, the decoding of the status of the probe, presented at the response phase, as pointing towards either the position of the target (hit/miss) or the distractor (false alarm/correct rejection), was also significant (56.1 ± 12.9%; *P* = 0.0099; see Fig. 6C). Notably, to avoid any effect due to a single visual stimulus at the sample phase we excluded from this analysis all the trials belonging to the “without distractor” condition.

All the results of these analyses were replicated when including the vaguely seen trials (i.e. PAS = 2): decoding of the target side during the sample and the delay (62.8 ± 14.6%; *P* < 0.0001; and 65.6 ± 12.3%; *P* < 0.0001; respectively), decoding of the distractor during the sample (56.3 ± 5.3%; *P* = 0.0001) but not during the delay (52.3 ± 6.7%; *P* = 0.0835), and decoding of the probe type (57.2 ± 12.2%; *P* = 0.0025).

##### Nonconscious trials

Decoding of the target side, irrespective of the presence or absence of the distractor, was not significant during the sample or delay phase (50.1 ± 5.3%; *P* = 0.32; and 49.4 ± 5.0%; *P* = 0.48, respectively; see Fig. 6C), thus providing no evidence for selective prioritization of the target. On the contrary, decoding of the presence vs. absence of the distractor was significant during the sample and delay phase (55.8 ± 5.1%; *P* < 0.0001; and 51.9 ± 5.6%; *P* = 0.022; see Fig. 6C), demonstrating that information of the distractor was encoded and maintained. However, the decoding accuracy of the presence vs. absence of the distractor did not correlate with the behavioral performance in presence of the distractor (i.e. hits—false alarms; r_28_ = −0.23, *P* = 0.21). Moreover, to certify the maintenance of the target, we ran a control analysis in which we decoded the target (without distractor) vs. the absent condition during the delay phase. This control analysis revealed that target presence was decodable well above chance (55.1 ± 5.9%; *P* < 0.0001), confirming the effective maintenance of the nonconscious target. By itself, decoding of the distractor presence is consistent with the behavioral pattern in both Experiments 1 and 2. In Experiment 1 there was no evidence of distractor interference, whereas in Experiment 2 the distractor location was remembered, but possibly due to the cue being presented nonconsciously and thus not tagged as a “distractor,” leading to a significant amount of false alarms.

To arbitrate between these 2 possibilities, we decoded the status of the probe, presented at the response phase, as pointing towards either the position of the target (hit/miss) or the distractor (false alarm/correct rejection). To avoid any effect due to a single visual stimulus at the sample phase we excluded from this analysis all the trials belonging to the “without distractor” condition. If information of the distractor is purposefully maintained to minimize false alarms, there should be a different pattern of brain activity when the probe is pointing to the position of the target compared with the position of the distractor. Alternatively, distractor information is not successfully filtered out, as in Experiment 2, and the same response-related brain activity would be expected for “target-match” and “distractor-match” probes (i.e. no significant decoding). Decoding of the probe as pointing towards the position of the target vs. distractor was significant (52.8 ± 8.3%; *P* = 0.020; see Fig. 6C), indicating that distractor information was maintained and tagged appropriately in relation to the task. Again, the decoding accuracy of the probe as pointing towards the position of the target vs. distractor did not correlate with the behavioral performance in presence of the distractor (i.e. hits—false alarms; *r*_28_ = 0.07, *P* = 0.73).

Although the motor cortex was excluded from the searchlight (see Fig. 6A), we wanted to ensure that decoding of probe status was not confounded by the specific finger used to respond. We therefore tried to decode the “yes/no” response during the Absent condition (i.e. false alarms vs. correct rejection in the Absent condition, where participants used the index and long finger, respectively). This decoding was not significant (49.5 ± 9.0%; *P* = 0.59), showing that the finger used to respond did not confound the classification performance of the probe type. Moreover, to exclude the possibility that decoding of probe status was confounded by a skewed distribution in terms of which location the probe was pointing at, which could cause a systematic difference in perceptual input, we performed a control analysis where the same probe-location distribution as in the original analysis was recreated, but where the probe status (as pointing to the target or distractor location) was counterbalanced, to specifically decode differences in the spatial distribution. Also, this decoding was not significant (50.2 ± 5.9%; *P* = 0.39), showing that the perceptual input was not confounding the decoding of probe status.

Lastly, we performed an additional control analysis to rule out the possibility that the presence of spurious nonconscious trials that may have been partially seen but mistakenly labeled as nonconscious (PAS = 1) could have driven the decoding of the nonconscious trials. To this end, we randomly split the absent trials into 2 bins and added a portion of conscious trials in one of the bins. As an estimate of erroneous PAS responses, we used the proportion of conscious trials with a PAS response < 3 (6.3%, see above). Classification of the “clean” vs. “contaminated” absent bins, during the sample phase, was not significant (50.8% ± 1.6, *P* = 0.28).

Together, the fMRI data show that task relevance can be assigned to nonconscious stimuli, but rather than filtering out distracting information, it seems that both the location of the target and the distractor were encoded and maintained. Counterintuitively, this leads to lower percentage of false alarms for nonconscious (after adjusting for the guessing rate) compared with conscious trials.

## Discussion

In summary, nonconsciously presented information can be actively maintained in memory during a brief delay, as evidenced in all 3 experiments: a hit rate significantly above the guessing rate in Experiments 1 and 2, and a significant decoding of brain activity for Targets vs. Absent trials as well as With vs. Without Distractor trials during the delay phase in Experiment 3. Crucially, the status of a stimulus as being a “target” or a “distractor” can also be established for nonconsciously presented stimuli, as evidenced by a significant difference between hits and false alarms in Experiment 1, and a significant decoding of brain activity of target vs. distractor location during the probe phase in Experiment 3. Such status assignment seems to be contingent on conscious processing of the cue, as evidenced by the lack of a significant difference between hits and false alarms in Experiment 2 (although both hits and false alarms were significantly above the guessing rate). However, despite successful target/distractor assignment in Experiments 1 and 3, there was no evidence for a prioritization of target location, because a decoding of target location could not be achieved during distractor-present trials in Experiment 3. Such prioritization was only evident during conscious trials. These findings extend previous research supporting the maintenance of nonconscious information (Soto et al. 2011; Bergström and Eriksson 2014; Soto and Silvanto 2014; Bergström and Eriksson 2015; King et al. 2016; Trübutschek et al. 2017; Bergström and Eriksson 2018; Trübutschek et al. 2019a, 2019b), by demonstrating that they can be also discriminated on the basis of their task relevance for prospective purposes (Fuster and Bressler 2012).

Previous literature on working memory in presence of distraction suggests that performance does not simply rely on working-memory capacity (Luck and Vogel 2013). On the contrary, it appears to be strongly affected by the ability to efficiently ignore distractors, and prioritize task-relevant information within the limited capacity of working memory (Vogel et al. 2005; McNab and Klingberg 2008; Fukuda and Vogel 2009; Gaspar et al. 2016; Liesefeld et al. 2020; Lorenc et al. 2021). The MVPA results from Experiment 3 indicate that the specific process used to deal with the task-irrelevant distractor was contingent on the conscious or nonconscious nature of the stimuli. For conscious stimuli, the brain activity patterns indicated selective prioritization and maintenance of the target, and filtering of the distractor, consistent with previous research (Vogel et al. 2005; Kuo et al. 2009; Simpson et al. 2011; Vossel et al. 2012). However, despite the apparent prioritization of the target, distractor interference was observed. Speculatively, distractor interference may in this case be a result of repetition suppression or other forms of memory that can also affect performance during working-memory tasks (e.g. Jiang et al. 2000).

The picture entirely changed when the stimuli were nonconscious. Here, the location of the target was not decodable during either the sample or delay phase, although the maintenance of the target was decodable when presented without a distractor (“target alone” vs. “target absent”). Moreover, the decoding of distractor presence was significant during both phases. These results together suggest encoding and maintenance of target and distractor. Combined with the significant decoding of probe type (i.e. as pointing towards the target or the distractor) during the probe phase, this indicates that not only target and distractor were maintained, but also the information related to task-relevance of the 2 shapes. Moreover, counterintuitively, the maintenance of target and distractor locations was combined with a nonsignificant rate of false alarms relative to the guessing rate during “absent” trials—a qualitatively different pattern compared with conscious trials.

These results confirmed, as observed in Experiment 1, the identification of nonconscious task-relevant information. Indeed, the decoding of the distractor presence during the delay, together with the decoding of the target when presented without a distractor during the delay (“target alone” vs. “target absent”), supports endogenous sustained attention to both target and distractor information during the maintenance of nonconscious information and it is in line with previous literature indicating sustained brain activity during the maintenance of nonconscious information (Bergström and Eriksson 2014; King et al. 2016; Bergström and Eriksson 2018; but see, Trübutschek et al. 2019b, for “activity-silent” nonconscious maintenance). Moreover, the better-than-chance decoding of the probe type extends these previous findings because it demonstrates that not only the 2 shapes were actively maintained in memory, but also their respective role in relation to the task. However, the current data shows selective prioritization of task-relevant information at both the encoding and maintenance phases only when the stimuli were conscious, thus suggesting a mismatch between the mechanisms involved in the execution of working memory tasks, depending on the conscious/nonconscious nature of the stimuli, when dealing with distraction. The lack of attentional prioritization could be due to the low memory load (at most 2 locations, if including the location of the distractor) that never exceeded the typical working-memory capacity limit. Indeed, in these experiments, it was not mandatory to selectively prioritize the target and suppress the distractor to solve the task. The task could also be solved through encoding and maintenance of both target and distractor, where the role of the 2 shapes was discriminated and distractor information may have been purposefully used to avoid false alarms. This latter mechanism, despite being more capacity consuming, could have been a successful way to solve the nonconscious task, in this specific setting where there was a low number of elements to retain. With the current design, it is not clear which processes during nonconscious encoding allowed the discrimination between the target and the distractor shapes. Indeed, the correct identification of the target could have resulted from “controlled” discrimination of the task-relevant information between the 2 shapes, or from “automatic” recognition of the task-relevant information derived from a perceptual match between cue and target shape. According to this latter interpretation, “automatic” mechanisms could have made it difficult to filter out the distractor information and could have led to the encoding and retention of both target and distractor information, where recognition of the target shape could have derived from the perceptual match between cue and target shape. A future step in the research about prioritization of nonconscious information could consist of increasing the amount of nonconscious distractors to evaluate if in a higher-load setting, identification and goal-directed use of task-relevant information would be still possible and if so, whether attentional prioritization to the target would be implemented nonconsciously.

Taken together, our data demonstrate that identification of task-relevant information is possible for nonconscious stimuli. However, it seems that the cue that defines task relevance must be visible to allow such identification of task-relevant information (Experiment 2). The requirement of a visible cue seems inconsistent with previous research showing top-down attentional effects when a spatial cue was nonconscious in a blindsight patient (Kentridge et al. 1999). There are several possible explanations for this discrepancy. It may be related to the fact that the current task relies on feature-based, as opposed to spatial, attention. Although feature-based attention has been demonstrated for nonconscious stimuli (Woodman and Luck 2003; Kanai et al. 2006; Tapia et al. 2010), attention was in these previous studies manipulated using conscious cues or by instruction, whereas the cue itself was nonconscious in Experiment 2. Thus, it is possible that feature-based top-down attention requires conscious experience of the cue/modulator whereas spatial top-down attention may not. Alternatively, the lack of nonconscious selection in Experiment 2 may be due to the combination of presenting both the cue and the target nonconsciously. As far as we can tell, this is the first experiment where both cue and target are presented nonconsciously. A third possibility is that top-down attention cannot here be elicited by nonconscious stimuli that are suppressed and thus is expected to give rise to a greatly weakened signal, whereas blindsight may constitute a special case where the nonconscious signal may be unusually strong. Lastly, a fourth possibility is in line with the “automatic” recognition of the target. According to this interpretation, both shapes could have been “automatically” encoded, but the target recognition could have required conscious perception of the cue shape to allow discrimination of the 2 shapes based on the “pattern match” between cue and target shape.

Although more sensitive than univariate analysis, MVPAs present also some limitations, related to their nondirectionality. MVPAs allow determining which kind of information is detectable in the brain, and permit investigating whether different conditions elicit distinguishable patterns of brain activity, but, they do not provide information about which of the 2 compared conditions drove the effect. An example of this limitation is the control analysis for the decoding of the target side in the conscious trials (see fMRI results of the Conscious trials). This control analysis was performed to exclude that decoding of the side of the target during the sample phase of the conscious trials could have derived from simpler perceptual processes, due to the presence of trials in which the visual stimulation was on a single side of the screen. The control analysis was restricted to those trials in which target and distractor were located on the opposite side. It is worth to note that, although counterintuitive, the better-than-chance decoding of the target side in this control analysis could have been significant also if the distractor (and not the target) was prioritized. However, the MVPA of the distractor presence during the delay phase of the conscious trials was nonsignificant, thus suggesting that the target’s location rather than distractor’s location, was attended. This pattern of brain activity during the delay phase, combined with the better-than-chance decoding of the target side during the sample and delay phase, supports attentional prioritization of the conscious target, despite the limitations due to the MVPAs.

A crucial aspect of the current study is the classification of trials as involving conscious perception of the sample stimuli or not. To this end we have relied on the PAS, which is a subjective measure of consciousness. Subjective and objective measures of consciousness have different pros and cons, and there is no consensus on which is preferable. Whereas subjective measures are vulnerable to response bias, objective measures may lead to a misinterpretation of behavior driven by nonconscious information as indicating consciousness, and the construct validity of objective measures is contentious. Consistently across the 3 experiments, participants reported to have seen something (PAS > 1) on about 10% of all “Absent” trials (i.e. trials where no sample stimulus was presented), demonstrating that they did not have a conservative criterion for reporting awareness. In addition, the current finding of a qualitatively different pattern in how distractor information is handled during conscious and nonconscious trials lend support to the PAS ratings as a veridical measure of consciousness. It is also noteworthy that task performance was at chance level for nonconscious trials during Experiment 3, and perception of the memory samples would thereby be considered nonconscious also by objective criteria.

To conclude, the current results provide support for 2 key components of working memory also when the memorandum is nonconscious: identification and sustained maintenance of task-relevant information. These findings suggest a neurocognitive construct that goes beyond a simple form of nonconscious short-term memory (Persuh et al. 2018). However, selective prioritization of task-relevant information was exclusively observed with conscious stimuli, thus indicating qualitatively different mechanisms involved during the execution of conscious and nonconscious working memory tasks, in presence of distraction. Whether or not the mechanisms underlying such nonconscious task performance should be classified as “working” memory remains unclear.

## Supplementary Material

Supplementary_material_bhac208
