# Supplementary material for: Nonconscious information can be identified as task-relevant but not prioritized in working memory
Source: Cereb Cortex. 2022 Jun 6;33(5):2287–301. doi: 10.1093/cercor/bhac208 (PMC9977358; doi:10.1093/cercor/bhac208)
Supplement: Supplementary_material_bhac208 [file supplementary_material_bhac208.docx]

**Supplementary Table 1** – Summary of the average number of trials, divided by PAS response and by condition (conscious/non-conscious/absent presentation, and probe type) in Experiment 1. The cells marked in bold indicate the final percentage of trials included in the analyses.

| **Experiment 1** | | | | | | | | | |
| --- | --- | --- | --- | --- | --- | --- | --- | --- | --- |
|  | Conscious trials | | | | | | | | |
|  | Target-match  (N = 31.8 ± 0.8) | | | Non-match  (N = 15.8 ± 0.5) | | | Distractor-match  (N = 15.9 ± 0.3) | | |
|  | PAS=1 | PAS=2 | **PAS=3** | PAS=1 | PAS=2 | **PAS=3** | PAS=1 | PAS=2 | **PAS=3** |
| Mean | 2.9 % | 3.2 % | **93.9 %** | 4.0 % | 3.0 % | **93.0 %** | 2.6 % | 4.3 % | **93.0** % |
| SD | 5.0 % | 7.2 % | **10.3 %** | 7.3 % | 8.5 % | **14.0 %** | 5.7 % | 9.6 % | **11.4** % |
|  | | | | | | | | | |
|  | Non-conscious trials | | | | | |  |  |  |
|  | Target-match  (N = 110.7 ± 4.2) | | | Distractor-match  (N = 110.7 ± 4.2) | | |  |  |  |
|  | **PAS=1** | PAS=2 | PAS=3 | **PAS=1** | PAS=2 | PAS=3 |  |  |  |
| Mean | **70.9 %** | 27.5 % | 1.6 % | **71.7 %** | 27.1 % | 1.2 % |  |  |  |
| SD | **10.9 %** | 10.7 % | 2.3 % | **11.4 %** | 11.4 % | 1.9 % |  |  |  |
|  | | | | | | |  |  |  |
|  | Absent trials  (N = 23.7 ± 1.0) | | |  |  |  |  |  |  |
|  | **PAS=1** | PAS=2 | PAS=3 |  |  |  |  |  |  |
| Mean | **89.7 %** | 10.1 % | 0.1 % |  |  |  |  |  |  |
| SD | **12.9 %** | 12.8 % | 0.8 % |  |  |  |  |  |  |
|  | | | | | | | | | |
| **Experiment 2** | | | | | | | | | |
|  | Conscious trials | | | | | | | | |
|  | Target-match  (N = 32.0 ± 0.2) | | | Non-match  (N = 16.0 ± 0.2) | | | Distractor-match  (N = 16.0 ± 0.2) | | |
|  | PAS=1 | PAS=2 | **PAS=3** | PAS=1 | PAS=2 | **PAS=3** | PAS=1 | PAS=2 | **PAS=3** |
| Mean | 2.4 % | 2.2 % | **95.4 %** | 2.6 % | 1.7 % | **95.7 %** | 2.9 % | 1.9 % | **95.2 %** |
| SD | 5.5 % | 3.9 % | **7.3 %** | 5.4 % | 3.8 % | **6.8 %** | 5.1 % | 4.2 % | **7.8 %** |
|  | | | | | | | | | |
|  | Non-conscious trials | | | | | |  |  |  |
|  | Target-match  (N = 110.3 ± 4.5) | | | Distractor-match  (N = 110.7 ± 3.9) | | |  |  |  |
|  | **PAS=1** | PAS=2 | PAS=3 | **PAS=1** | PAS=2 | PAS=3 |  |  |  |
| Mean | **70.7 %** | 28.7 % | 0.7 % | **71.4 %** | 27.5 % | 1.0 % |  |  |  |
| SD | **14.9 %** | 14.9 % | 1.6 % | **14.4 %** | 14.4 % | 2.5 % |  |  |  |
|  | | | | | | |  |  |  |
|  | Absent trials  (N = 23.6 ± 1.2) | | |  |  |  |  |  |  |
|  | **PAS=1** | PAS=2 | PAS=3 |  |  |  |  |  |  |
| Mean | **89.2 %** | 10.8 % | 0.0 % |  |  |  |  |  |  |
| SD | **14.5 %** | 14.5 % | 0.0 % |  |  |  |  |  |  |

**Supplementary Table 2** – Summary of the average number of trials, divided by PAS response and by condition (conscious/non-conscious/absent presentation, left/right target, target alone/target with distractor, and probe type) in Experiment 3. The cells marked in bold indicate the final percentage of trials included in the analyses.

| **Experiment 3** | | | | | | | | | | | | | | | |
| --- | --- | --- | --- | --- | --- | --- | --- | --- | --- | --- | --- | --- | --- | --- | --- |
|  | Conscious trials – Target on the left | | | | | | | | | | | | | | |
|  | Target alone | | | | | | Target with distractor | | | | | | | | |
|  | Target-match  (N = 5.9 ± 0.3) | | | Non-match  (N = 5.9 ± 0.3) | | | Target-match  (N = 8.9 ± 0.3) | | | Non-match  (N = 5.9 ± 0.3) | | | Distractor-match  (N = 3.0 ± 0.0) | | |
|  | Pas=1 | Pas=2 | **Pas=3** | Pas=1 | Pas=2 | **Pas=3** | Pas=1 | Pas=2 | **Pas=3** | Pas=1 | Pas=2 | **Pas=3** | Pas=1 | Pas=2 | **Pas=3** |
| Mean  (%) | 2.9 | 1.1 | **96.0** | 6.9 | 2.3 | **90.8** | 2.4 | 1.9 | **95.8** | 4.0 | 1.2 | **94.8** | 7.8 | 1.1 | **91.1** |
| SD | 6.6 | 4.2 | **7.4** | 11.5 | 7.5 | **16.1** | 5.8 | 4.2 | **7.7** | 8.6 | 4.7 | **9.3** | 18.9 | 6.1 | **19.4** |
|  | | | | | | | | | | | | | | | |
|  | Conscious trials – Target on the right | | | | | | | | | | | | | | |
|  | Target alone | | | | | | Target with distractor | | | | | | | | |
|  | Target-match  (N = 5.9 ± 0.3) | | | Non-match  (N = 5.9 ± 0.4) | | | Target-match  (N = 8.8 ± 0.5) | | | Non-match  (N = 3.0 ± 0.2) | | | Distractor-match  (N = 5.9 ± 0.3) | | |
|  | Pas=1 | Pas=2 | **Pas=3** | Pas=1 | Pas=2 | **Pas=3** | Pas=1 | Pas=2 | **Pas=3** | Pas=1 | Pas=2 | **Pas=3** | Pas=1 | Pas=2 | **Pas=3** |
| Mean  (%) | 4.9 | 0.6 | **94.6** | 5.2 | 1.7 | **93.1** | 4.2 | 1.5 | **94.3** | 4.4 | 1.1 | **94.4** | 5.6 | 1.7 | **92.8** |
| SD | 15.3 | 3.0 | **15.4** | 9.3 | 5.1 | **10.6** | 7.6 | 4.0 | **10.2** | 14.5 | 6.1 | **15.4** | 9.1 | 6.7 | **15.4** |
|  | | | | | | | | | | | | | | | |
|  | Non-conscious trials **-** Target on the left | | | | | | | | | | | |  |  |  |
|  | Target alone | | | | | | Target with distractor | | | | | |  |  |  |
|  | Target-match  (N = 11.7 ± 1.1) | | | Non-match  (N = 11.7 ± 1.0) | | | Target-match  (N = 17.6 ± 0.9) | | | Distractor-match (N = 17.5 ± 1.3) | | |  |  |  |
|  | **Pas=1** | Pas=2 | Pas=3 | **Pas=1** | Pas=2 | Pas=3 | **Pas=1** | Pas=2 | Pas=3 | **Pas=1** | Pas=2 | Pas=3 |  |  |  |
| Mean  (%) | **86.9** | 13.1 | 0.0 | **85.2** | 14.8 | 0.0 | **82.7** | 16.4 | 0.9 | **82.3** | 17.3 | 0.4 |  |  |  |
| SD | **13.4** | 13.4 | 0.0 | **17.3** | 17.3 | 0.0 | **14.3** | 14.5 | 2.2 | **17.3** | 17.0 | 1.4 |  |  |  |
|  | | | | | | | | | | | | |  |  |  |
|  | Non-conscious trials - Target on the right | | | | | | | | | | | |  |  |  |
|  | Target alone | | | | | | Target with distractor | | | | | |  |  |  |
|  | Target-match  (N = 11.6 ± 0.8) | | | Non-match  (N = 11.6 ± 0.7) | | | Target-match  (N = 17.7 ± 0.8) | | | Distractor-match (N = 17.7 ± 0.6) | | |  |  |  |
|  | **Pas=1** | Pas=2 | Pas=3 | **Pas=1** | Pas=2 | Pas=3 | **Pas=1** | Pas=2 | Pas=3 | **Pas=1** | Pas=2 | Pas=3 |  |  |  |
| Mean  (%) | **83.8** | 15.4 | 0.8 | **80.9** | 17.6 | 1.5 | **76.0** | 23.3 | 0.7 | **79.5** | 19.9 | 0.6 |  |  |  |
| SD | **16.9** | 17.0 | 2.5 | **13. 7** | 12.9 | 3.5 | **16.8** | 16.5 | 2.4 | **14.3** | 13.9 | 1.8 |  |  |  |
|  | | | | | | | | | | | | |  |  |  |
|  | Absent trials  (N = 41.0 ± 2.2) | | |  |  |  |  |  |  |  |  |  |  |  |  |
|  | **PAS=1** | PAS=2 | PAS=3 |  |  |  |  |  |  |  |  |  |  |  |  |
| Mean  (%) | **90.1** | 9.5 | 0.4 |  |  |  |  |  |  |  |  |  |  |  |  |
| SD | **12.0** | 12.0 | 1.2 |  |  |  |  |  |  |  |  |  |  |  |  |

**Supplementary Figure 1 –** Behavioral performance for the working-memory task in the three experiments in the non-conscious and absent conditions. In the non-conscious (blue) condition, the violin plots show the percentage of hits when the probe pointed towards the correct location (Hit), the percentage of false alarms when the probe pointed towards the distractor’s position (FA distractor) or an empty position (FA non-match). For the absent (gray) condition the behavioral performance is the percentage of false alarms (“yes” responses, FA absent). In all violin plots, median and mean are represented by the white dot and the horizontal black line, respectively.

**
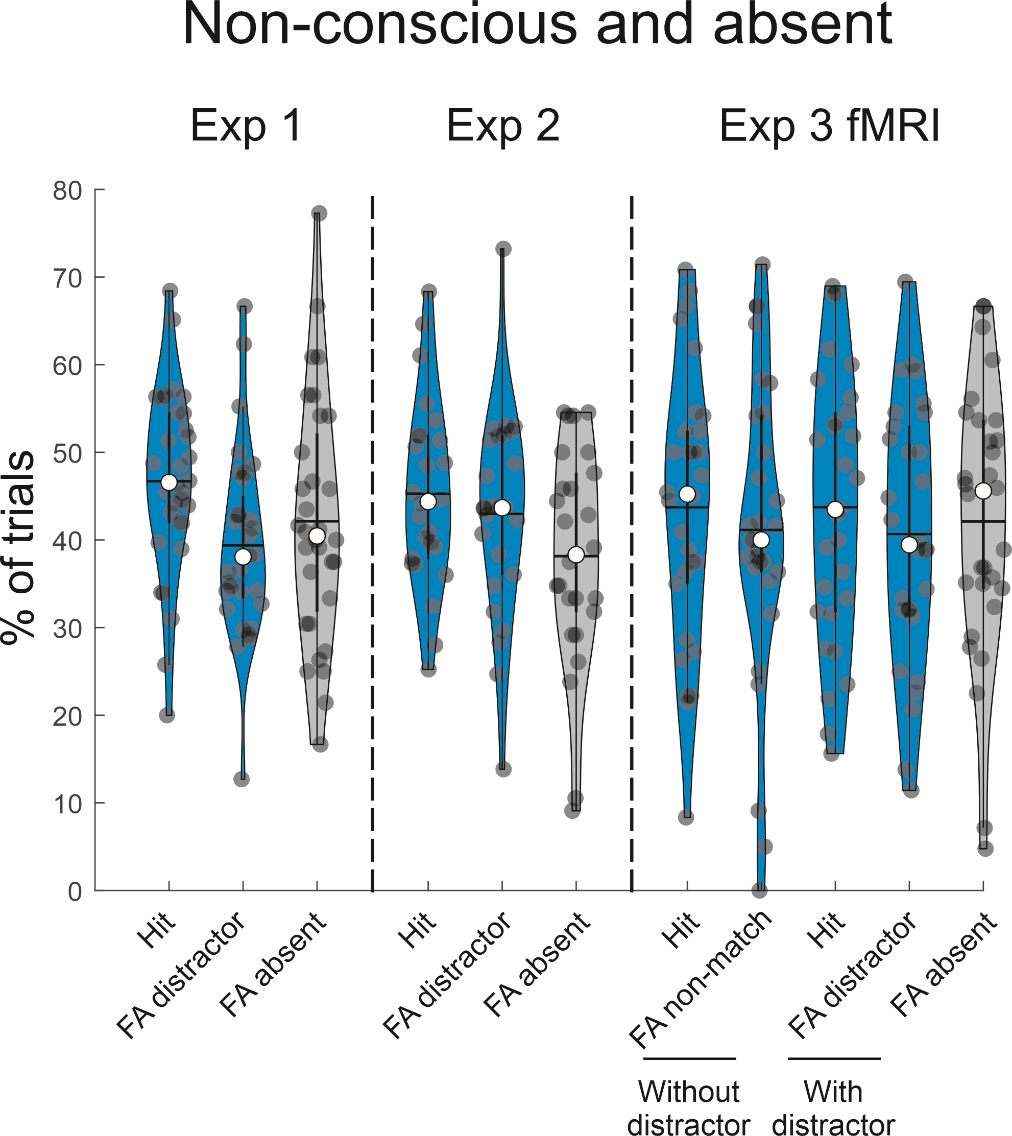
**
